# A new detection model of microaneurysms based on improved FC-DenseNet

**DOI:** 10.1038/s41598-021-04750-2

**Published:** 2022-01-19

**Authors:** Zhenhua Wang, Xiaokai Li, Mudi Yao, Jing Li, Qing Jiang, Biao Yan

**Affiliations:** 1 College of Information Science, Shanghai Ocean University, Shanghai, 201306 China; 2 The Affiliated Eye Hospital, Nanjing Medical University, Nanjing, 211166 China; 3 The Affiliated Sixth People’s Hospital, Shanghai Jiaotong University, Shanghai, 200233 China; 4 Eye Institute, Eye and ENT Hospital, Shanghai Medical College, Fudan University, Shanghai, 200030 China

**Keywords:** Retinal diseases, Data processing

## Abstract

Diabetic retinopathy (DR) is a frequent vascular complication of diabetes mellitus and remains a leading cause of vision loss worldwide. Microaneurysm (MA) is usually the first symptom of DR that leads to blood leakage in the retina. Periodic detection of MAs will facilitate early detection of DR and reduction of vision injury. In this study, we proposed a novel model for the detection of MAs in fluorescein fundus angiography (FFA) images based on the improved FC-DenseNet, MAs-FC-DenseNet. FFA images were pre-processed by the Histogram Stretching and Gaussian Filtering algorithm to improve the quality of FFA images. Then, MA regions were detected by the improved FC-DenseNet. MAs-FC-DenseNet was compared against other FC-DenseNet models (FC-DenseNet56 and FC-DenseNet67) or the end-to-end models (DeeplabV3+ and PSPNet) to evaluate the detection performance of MAs. The result suggested that MAs-FC-DenseNet had higher values of evaluation metrics than other models, including pixel accuracy (*PA*), mean pixel accuracy (*MPA*), precision (*Pre*), recall (*Re*), F1-score (*F1*), and mean intersection over union (*MIoU*). Moreover, MA detection performance for MAs-FC-DenseNet was very close to the ground truth. Taken together, MAs-FC-DenseNet is a reliable model for rapid and accurate detection of MAs, which would be used for mass screening of DR patients.

## Introduction

Retinal microaneurysms (MAs) are defined as the small swelling of tiny blood vessels, which mainly locate in the inner nuclear layer and deep capillary layer^1^. MAs often occur as the early clinical signs of retinal diseases, such as diabetic retinopathy (DR) and retinal vein occlusions. The number and turnover of retinal MAs are considered as the indicators to assess the presence, severity, and progression risk of retinopathy. Thus, periodic detection of MAs is required for the early diagnosis of retinopathy. MAs can be identified by several imaging technologies, including color fundus photography, fundus fluorescein angiography (FFA), and optical coherence tomography angiography (OCTA). Clinically, FFA is well-recognized as an important standard to visualize retinal vasculature and is routinely used to describe the subtle vascular alterations^2^.

FFA is highly sensitive and demonstrates MAs as the hyperfluorescent dots in the early phase. It is an important imaging modality, which can capture images after the intravenous injection of fluorescein dye^3^. With the increasing amount of FFA images that require for analysis, manual detection and quantification of MAs have become the labor-intensive and time-consuming jobs^4^. In addition, manual detection of MAs is subjective and error-prone, which may cause poor reproducibility^5^. Thus, an automated detection method is urgently required for the accurate detection of MAs in FFA images.

Recently, the development of MA detection methods have become a hot topic in the field of ophthalmic study. Some models based on the neural network have been used for MA detection. CNN and ResNet could obtain higher-level features from the upper layer and give up the features of the lower layer, but these model may loss parts of small-size targets^6,7^. DeeplabV3+and PSPNet use spatial pyramid pooling module to further extract contextual information and improve the detection accuracy of small-size targets, but they have misdetection and missed detection problems for MA detection^8,9^. Some improvements on neural network have been used for MA detection. Mazlan et al. proposed a detection method for using H-maxima and thresholding technique^10^. Sarhan et al. proposed a two-stage deep learning approach for MA segmentation using the multiple scales of the input with selective sampling and embedding triplet loss^11^. Kou et al. proposed an architecture for U-Net obtained by combining the deep residual model and recurrent convolutional operations into U-Net^12^. Reguant et al. proposed an unsupervised method for DR detection based on CNN^13^. González-Gonzalo et al. proposed a deep visualization method based on the unsupervised selective inpainting^14^. However, MAs usually have low contrast and tiny size. The pixels of MAs are often similar as the pixels of blood vessels. Thus, it still required to develop novel methods to further improve the detection accuracy of MAs.

In this study, we proposed a novel model for detecting MAs in FFA images based on the improved FC-DenseNet, MAs-FC-DenseNet. This model showed advantage over other FC-DenseNet models (FC-DenseNet56 and FC-DenseNet67) or the end-to-end models (DeeplabV3+and PSPNet) for MA detection. MAs-FC-DenseNet may become a promising method for rapid and accurate detection of MAs during mass screening of DR patients.

## Materials and methods

### Proposed MA detection model, MAs-FC-DenseNet

**Figure 1:**
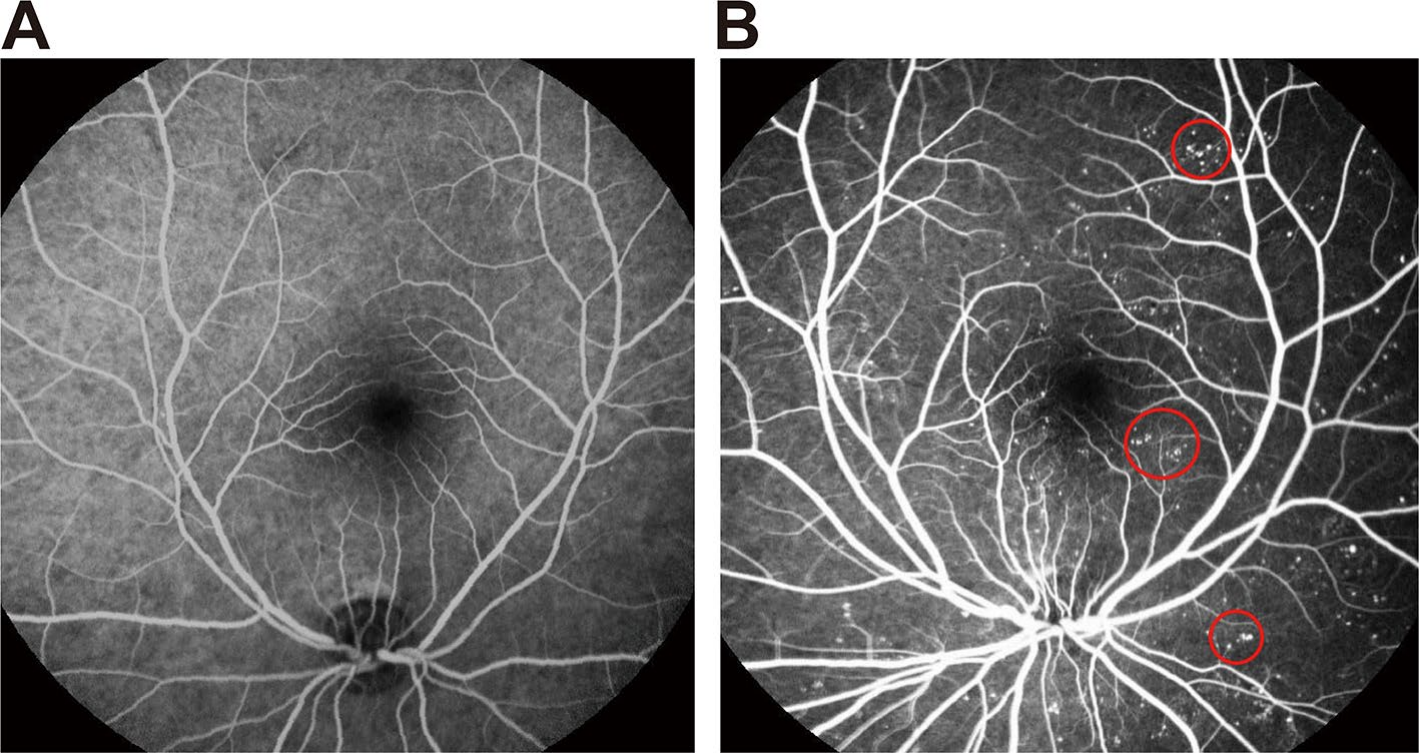
Normal FFA image and FFA image with Mas. (**A**) Normal FFA image; (**B**) FFA image with MAs.

Normal FFA image and FFA image with MAs are shown in Fig. 1. MAs are the small white round spots in FFA image as shown in Fig. 1B.

**Figure 2:**
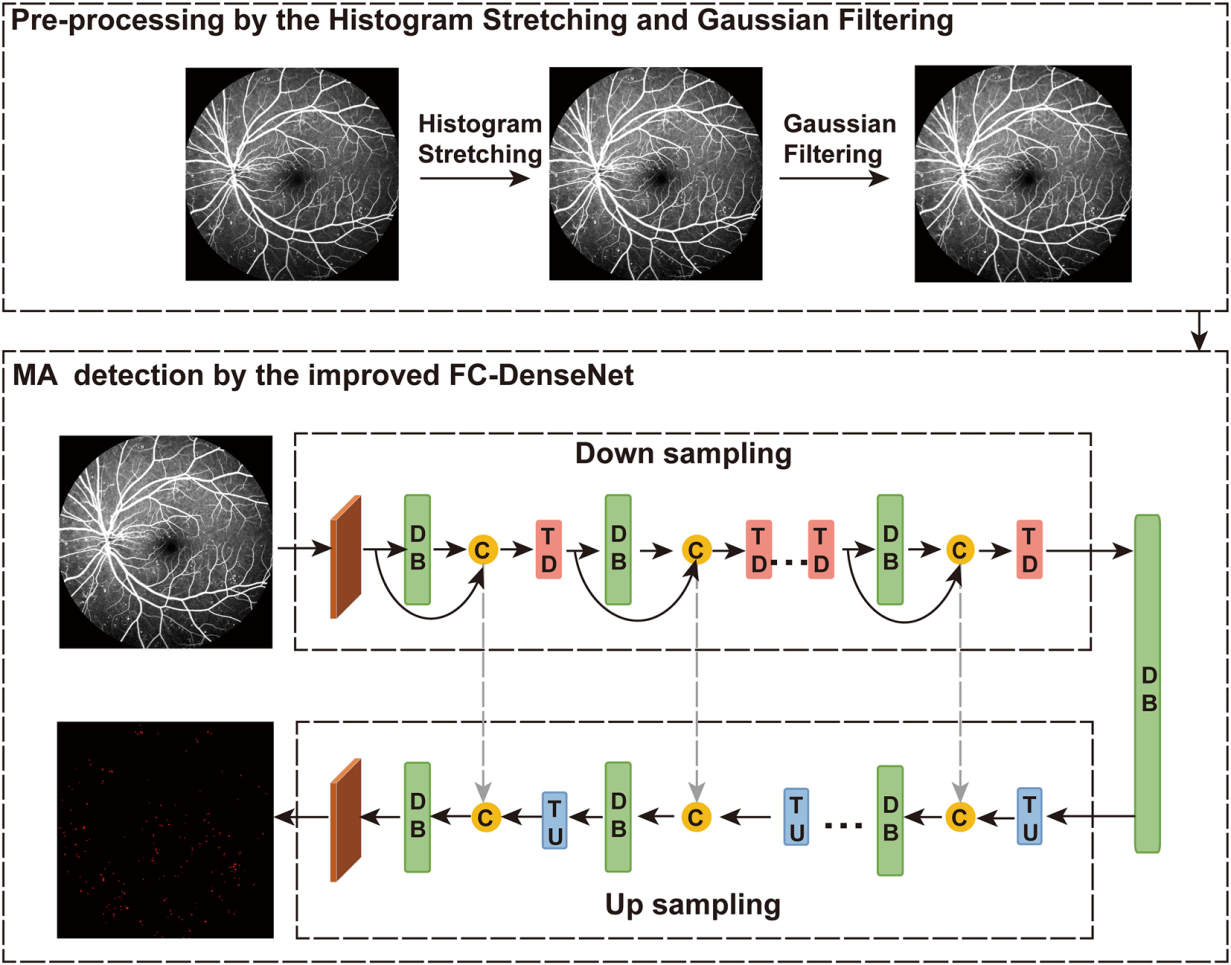
Flowchart of MAs-FC-DenseNet.

Figure 2 shows the flowchart of MAs-FC-DenseNet model, including the pre-processing of FFA images by the Histogram Stretching and Gaussian Filtering and MA detection by the improved FC-DenseNet.

### Pre-processing of FFA images by the histogram stretching and Gaussian filtering

FFA images were pre-processed to improve their contrast and reduce image noises. The contrast between MAs and the background was enhanced by the Histogram stretching. Histogram stretching could stretch the values of pixel from 0 to 255^15–17^. The noises of FFA images were then reduced by the Gaussian Filtering. Gaussian Filtering could remove the surrounding noises in the non-uniform retinal images^18–20^.

Histogram Stretching is expressed as

1 $$I_{{{\text{new}}}} = \left( {\frac{{G_{\max } - G_{\min } }}{{I_{\max } - I_{\min } }}} \right)\left( {I - I_{\min } } \right) + G_{\min }$$

where *I*_new_ is the new transformed image; *I*_max_ and *I*_min_ are the largest and smallest possible grey level value in the original image, respectively. *G*_max_ and *G*_min_ are the largest and smallest possible grey level value in the transformed image, respectively.

Gaussian Filtering is expressed as

2 $$G(x,y) = \frac{1}{{\sqrt {2\pi } \sigma }}\exp \left( { - \left( {x^{2} { + }y^{2} } \right){/2}\sigma^{{2}} } \right)$$

where *σ*^2^ is the variance of Gaussian Filtering; *l* is the size of the filter kernel.

### MA detection by the improved FC-DenseNet

At this step, MAs were detected by the improved Fully Convolutional DenseNet (FC-DenseNet)^21^. FC-DenseNet contains the down sampling path for extracting sparse semantic features and the up sampling path for restoring original resolution. The down sampling path consists of dense block (DB) layer and transition down (TD) layer^22^. The up sampling path consists of DB layer and transition up (TU) layer. DB layer is composed of batch normalization (BN)^23^, ReLU^24^, 3 × 3 convolution, and dropout with probability *p* = 0.2. TD layer is composed of BN, ReLU, 1 × 1 convolution, dropout with probability *p* = 0.2 and 2 × 2 maximum pooling. TU layer includes 3 × 3 transposed convolution with stride 2.

As shown in Fig. 2, the feature maps from the down sampling path were concatenated with the corresponding feature maps in the up sampling path. The connectivity pattern in the up sampling and the down sampling paths were different. In the down sampling path, the input to a dense block was concatenated with its output, leading to a linear growth of the number of feature maps, whereas in the up sampling path, it was not.

Due to the closeness of MAs to the vessels and low number of pixels belonging to MAs, it is difficult to accurately detect MAs. Here, we replaced the cross entropy loss of FC-DenseNet with the focal loss^25^. The focal loss could decrease the weight of the background and increase the weight of MAs. Thus, this model could increase the detection preformation of MA regions.

Here, the focal loss function is expressed as

3 $$FL(p_{t} ) = - \alpha_{t} (1 - p_{t} )^{\gamma } \log (p_{t} )$$

where *p*_*t*_ is probability of correct prediction for different categories. *α*_*t*_ and *γ* ≥ 0 are adjustable hyperparameters, which can be used to control the sharing weight of different samples to the total loss.

### Datasets

FFA image cohort was constructed with the collaboration of the Affiliated Eye Hospital of Nanjing Medical University, the first Affiliated Hospital of Soochow University, and Huai'An First People's Hospital. The dataset contains 1200 FFA images (768 × 868 pixels) from 1200 eyes of DR patients (age range 31–81 years old) who underwent FFA in three hospitals from August 2015 to December 2019. Each hospital provided 400 images. The operations were performed by the experienced clinicians using the Heidelberg Retina Angiograph (Heidelberg Engineering, Heidelberg, Germany). The dataset did not include the blurry or overexposed FFA images caused by the environmental factors or equipment materials. This study was approved by the Ethics Committee of the Affiliated Eye Hospital, Nanjing Medical University. The procedures adhered to the tenets of the Declaration of Helsinki. Written informed consents were obtained from all participants. Subsets of 960, 120 and 120 FFA images were randomly selected for training, validation and testing, respectively. Each FFA image was individually labeled by 3 experienced clinicians with more than 10-year clinical working experiences. Due to the limited human energy and occasional blurred images, some artificial deviations would inevitably occur during MA label. For these images, thorough rounds of discussion and adjudication were needed until full consensus was reached.

### Evaluation metrics

Six metrics, including pixel accuracy *(PA*), mean pixel accuracy (*MPA*), Precision (*Pre*), Recall (*Re*), F1-score (*F1*), and mean intersection over union (*MIoU*) were calculated to estimate the detection performance of MAs^26–29^.

4 $$PA{ = }\frac{{\sum\nolimits_{i = 0}^{k} {p_{ii} } }}{{\sum\nolimits_{i = 0}^{k} {\sum\nolimits_{j = 0}^{k} {p_{ij} } } }}$$

5 $$MPA{ = }\frac{1}{k + 1}\sum\limits_{i = 0}^{k} {\frac{{p_{ii} }}{{\sum\nolimits_{j = 0}^{k} {p_{ij} } }}}$$

6 $$Pre = \frac{TP}{{TP + FP}}$$

7 $$Re = \frac{TP}{{TP + FN}}$$

8 $$F1 = \frac{2 \times Pre \times Re}{{Pre + Re}}$$

9 $$MIoU{ = }\frac{1}{k + 1}\sum\limits_{i = 0}^{k} {\frac{{p_{ii} }}{{\sum\nolimits_{j = 0}^{k} {p_{ij} + \sum\nolimits_{j = 0}^{k} {p_{ji} - p_{ii} } } }}}$$

*TP*, *FP*, and *FN* denote the true positive region, false positive region, and false negative region, respectively. *k* indicates the labeling results of different classes, where *k* = 0 expressed as background class and *k* = 1 as MAs class. *p*_*ij*_ is the number of pixels of class *i* predicted to belong to class j, among $i,j \in \left[ {0,1} \right]$. *PA* is the overall pixel accuracy. *MPA* is the average pixel accuracy of MAs and background. *Pre* and *Re* are the proportion of real MAs in the samples predicted as MAs and the proportion of correct predictions in all MAs, respectively. F1-score (*F1*) is a balanced metric and determined by the precision and recall. Mean intersection over union (*MIoU*) is an accuracy assessment metric applied to measure the similarity between ground truth and prediction.

### Implementation

The hardware configuration used for the experiment were shown below: Ubuntu 16.04.4, 2GPUs, GPU NVIDIA Tesla P100 PCIE, and 1 GPU memory (16 GB). Software environment was Deep-learning framework Tensorflow1.8.0 and programming language python 3.6.

## Results

**Figure 3:**
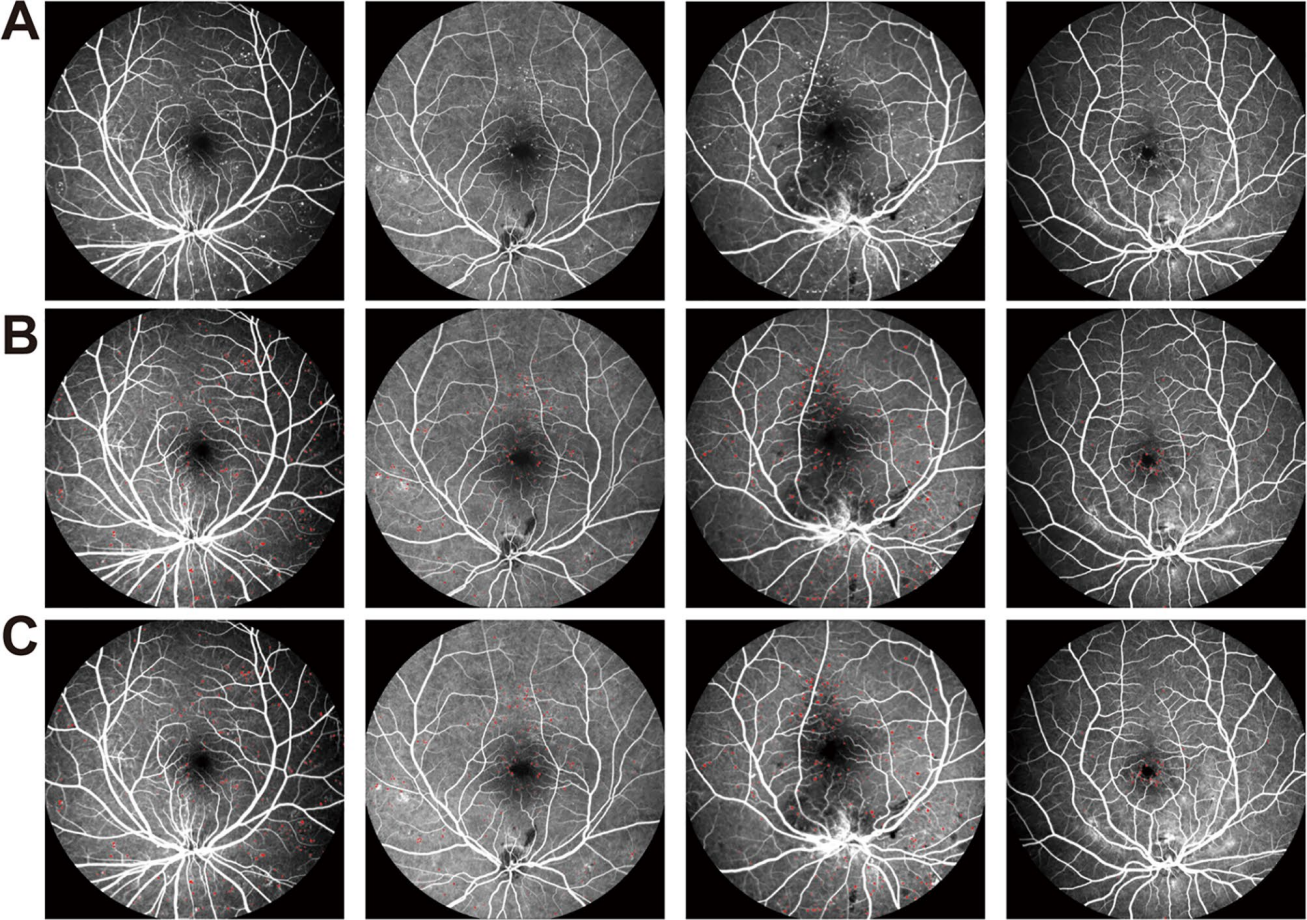
Detection results of MAs in FFA images. (**A**) Original FFA image; (**B**) detection results of MAs by MAs-FC-DenseNet; (**C**) ground truth.

The original FFA images, the detection results by MAs-FC-DenseNet, and the ground truth by the clinicians were shown in Fig. 3. To evaluate the detection performance for MAs, three comparison experiments were conducted. Experiment 1 was an ablation experiment. In experiment 2, MAs-FC-DenseNet was compared against other FC-DenseNet models including FC-DenseNet56 and FC-DenseNet67 to compare MA detection performance. In experiment 3, MAs-FC-DenseNet was compared against other end-to-end models, including DeeplabV3+ and PSPNet, to compare MA detection performance.

### Ablation experiment

**Figure 4:**
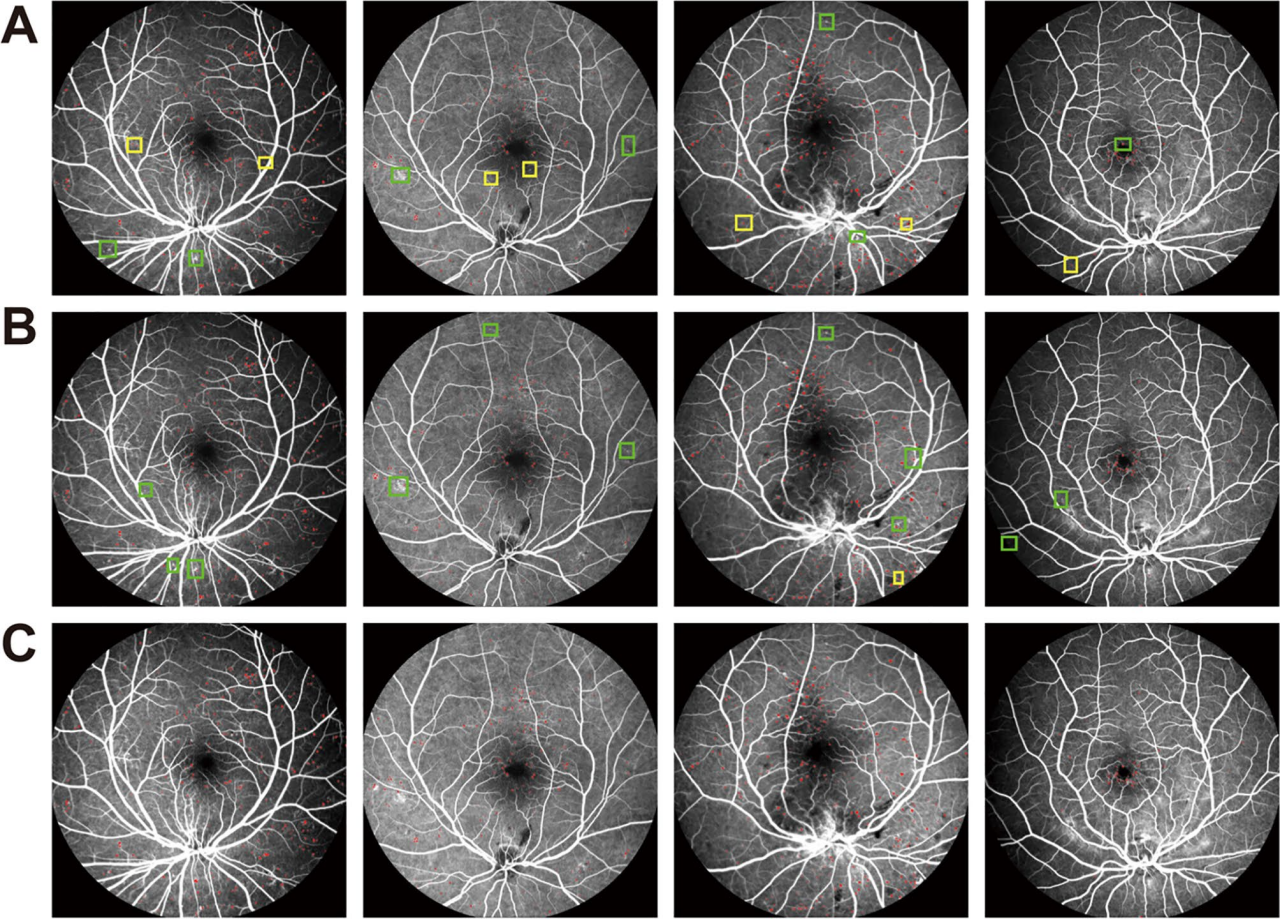
MA detection results in ablation experiment. (**A**) Pre-processing + FC-DenseNet103; (**B**) FC-DenseNet103 + Focal loss; (**C**) MAs-FC-DenseNet.

Figure 4 and Table 1 showed the comparison result of MA detection performance in the ablation studies, including FC-DenseNet103 with pre-processing (pre-processing + FC-DenseNet103), the improved FC-DenseNet103 without pre-processing (FC-DenseNet103 + Focal loss), and the proposed MAs-FC-DenseNet.

**Table 1:** Comparison of MA detection performance in ablation experiment.

Model	Evaluation metrics					
	*PA* (%)	*MPA* (%)	*Pre* (%)	*Re* (%)	*F*1 (%)	*MIoU* (%)
Pre-processing + FC-DenseNet103	99.95 ± 0.02	88.91 ± 0.13	77.84 ± 0.18	87.74 ± 0.09	82.20 ± 0.13	84.99 ± 0.09
FC-DenseNet103 + Focal loss	99.95 ± 0.02	90.61 ± 0.08	81.24 ± 0.16	77.18 ± 0.17	77.99 ± 0.18	82.42 ± 0.11
MAs-FC-DenseNet	99.97 ± 0.01	94.19 ± 0.04	88.40 ± 0.06	89.70 ± 0.05	88.98 ± 0.06	90.14 ± 0.05

As shown in Fig. 4 and Table 1, the models of FC-DenseNet103 with pre-processing or improved FC-DenseNet103 without pre-processing led to some missed and false detection of MAs. By contrast, MAs-FC-DenseNet reduced the missed and false detection regions and significantly enhanced the values of *PA*,* MPA*,* Pre*,* Re*,* F1*, and *MIoU*.

### Evaluation of MA detection performance of MAs-FC-DenseNet by comparing against other FC-DenseNet models

MAs-FC-DenseNet was compared against other FC-DenseNet models, including FC-DenseNet56 and FC-DenseNet67, to evaluate the detection performance of MAs.

**Figure 5:**
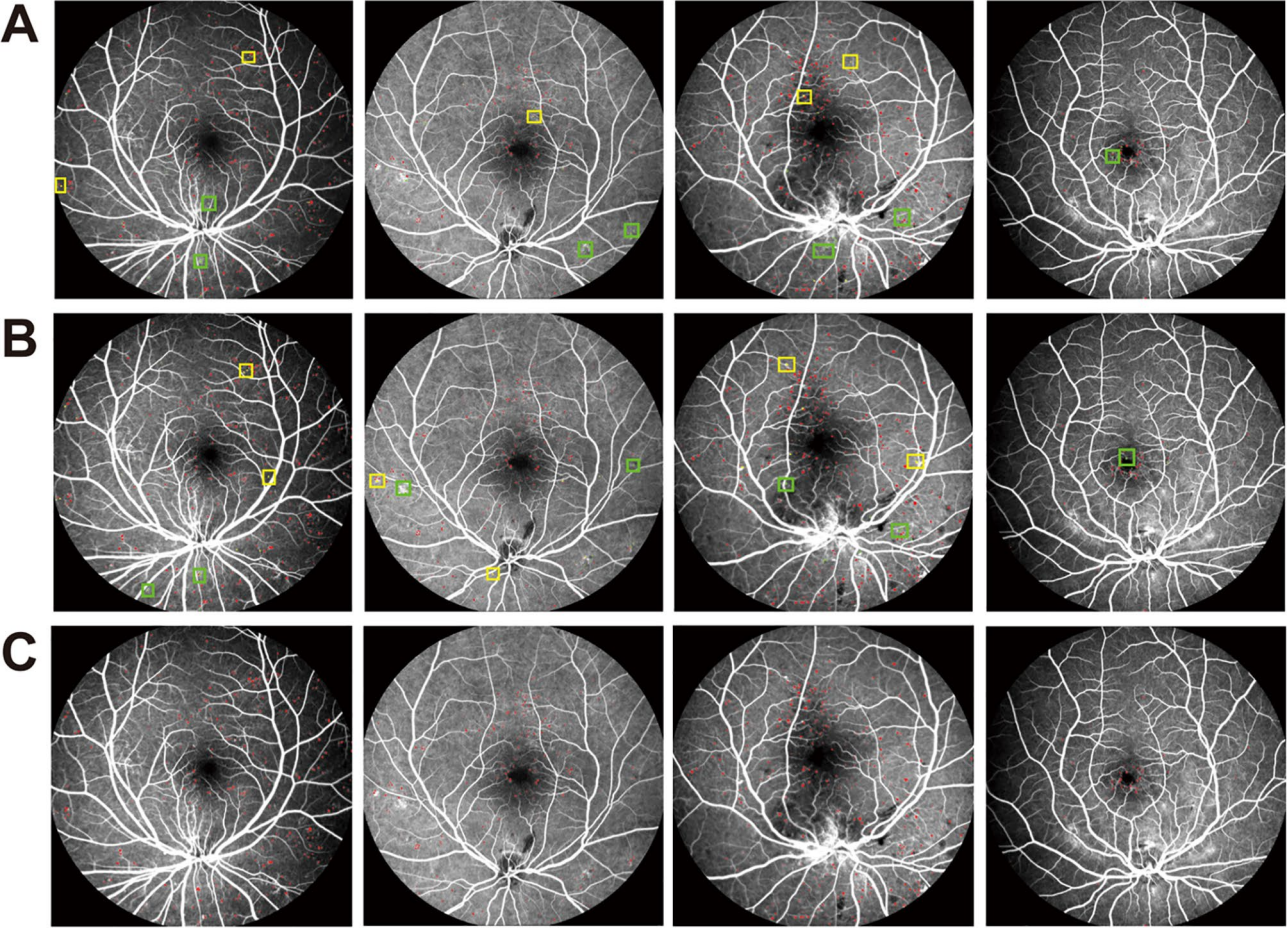
MA detection results by FC-DenseNet56, FC-DenseNet67, and MAs-FC-DenseNet. (**A**) FC-DenseNet56; (**B**) FC-DenseNet67; (**C**) MAs-FC-DenseNet.

As shown in Fig. 5 and Table 2, some MA regions were missed and falsely detected in FC-DenseNet56 and FC-DenseNet67 model. MA detection result of proposed MAs-FC-DenseNet achieved greater values of *PA*, *MPA*, *Pre*, *Re*, *F1*, and *MIoU*, which were 99.97% (0.01↑), 94.19% (2.82↑), 88.40% (5.67↑), 89.70% (8.76↑), 88.98% (7.86↑), and 90.14% (5.80↑), respectively.

**Table 2:** Comparison of MA detection performance by FC-DenseNet56, FC-DenseNet67, and MAs-FC-DenseNet.

Models	Evaluation metrics					
	*PA* (%)	*MPA* (%)	*Pre* (%)	*Re* (%)	*F*1 (%)	*MIoU* (%)
FC-DenseNet56	99.96 ± 0.02	88.74 ± 0.11	77.49 ± 0.17	80.87 ± 0.10	78.07 ± 0.16	82.30 ± 0.09
FC-DenseNet67	99.96 ± 0.02	91.37 ± 0.06	82.73 ± 0.09	80.94 ± 0.11	81.12 ± 0.10	84.34 ± 0.09
MAs-FC-DenseNet	99.97 ± 0.01	94.19 ± 0.04	88.40 ± 0.06	89.70 ± 0.05	88.98 ± 0.06	90.14 ± 0.05

### Evaluation of MA detection performance of MAs-FC-DenseNet by comparing against other end-to-end models

MAs-FC-DenseNet was compared against other end-to-end models, including DeeplabV3+ and PSPNet, to evaluate the detection performance of MAs.

**Figure 6:**
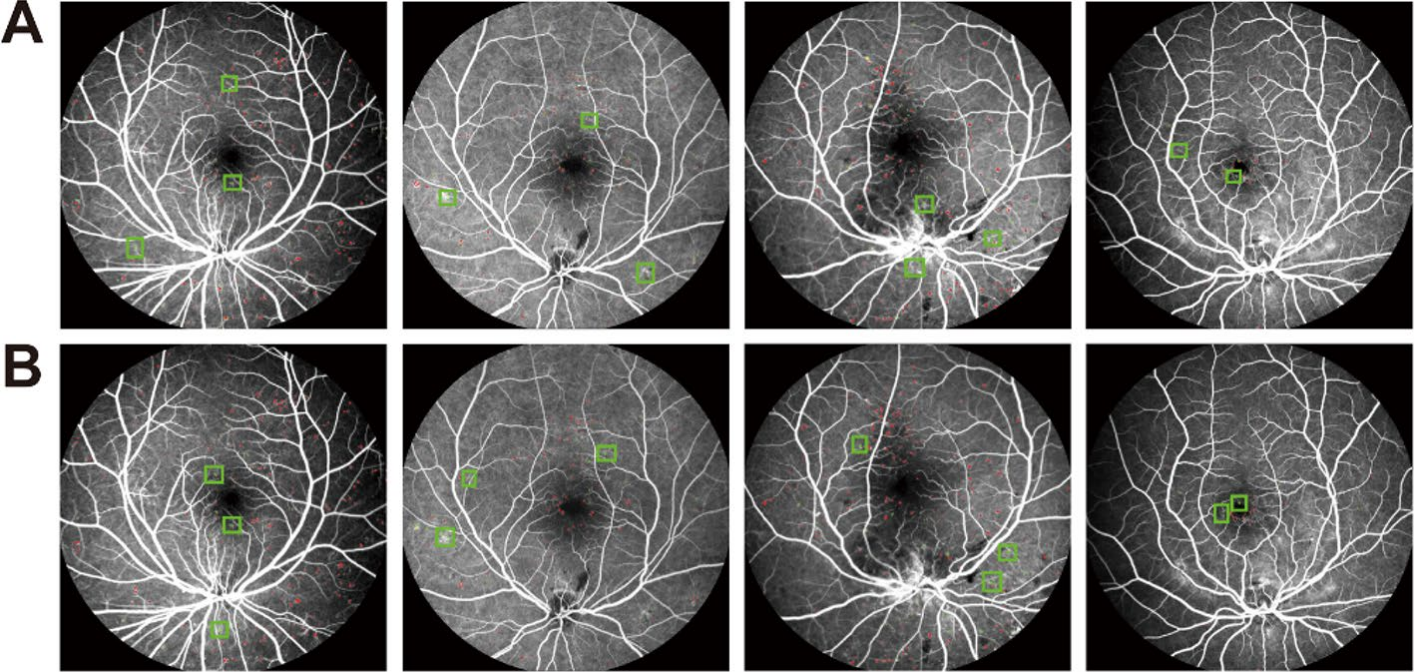
MA detection results by DeeplabV3+ and PSPNet. (**A**) DeeplabV3+; (**B**) PSPNet.

As shown in Fig. 6 and Table 3, the DeeplabV3+ model could not distinguish the boundaries of MAs and normal regions, which led to some false detection of MAs. As for PSPNet model, some MA regions were missed. MA detection result of proposed MAs-FC-DenseNet was very close to the ground truth. Moreover, MAs-FC-DenseNet had grater values of *PA*, *MPA*, *Pre*, *Re*, *F1*, and *MIoU* than that of other models.

**Table 3:** Comparison of MA detection performance by DeeplabV3+, PSPNet, and MAs-FC-DenseNet.

Models	Evaluation metrics					
	*PA* (%)	*MPA* (%)	*Pre* (%)	*Re* (%)	*F*1 (%)	*MIoU (%)*
DeeplabV3 +	99.91 ± 0.04	71.03 ± 0.19	42.08 ± 0.27	69.36 ± 0.19	51.12 ± 0.26	67.60 ± 0.21
PSPNet	99.93 ± 0.03	81.85 ± 0.14	63.73 ± 0.21	76.00 ± 0.12	66.96 ± 0.20	75.46 ± 0.18
MAs-FC-DenseNet	99.97 ± 0.01	94.19 ± 0.04	88.40 ± 0.06	89.70 ± 0.05	88.98 ± 0.06	90.14 ± 0.05

## Discussion

Microaneurysm (MA) is recognized as the first symptom of DR that leads to retinal blood injury. Detection of MAs within FFA images facilitates early DR detection and prevents vision loss^30^. However, MA is extremely small and its contrast to the surrounding background is very subtle, which make MA detection challenging. In this study, we proposed a novel model, MAs-FC-DenseNet, for the detection of MAs in FFA images. FFA images were pre-processed to enhance image quality by the Histogram Stretching and Gaussian Filtering. Improved FC-DenseNet model was then used to detect the deep features of MAs and enhance the detection accuracy of MAs.

Six metrics were used to evaluate the accuracy for MA detection, including pixel accuracy (*PA*), mean pixel accuracy (*MPA*), Precision (*Pre*), Recall (*Re*), F1-score (*F1*), and mean intersection over union (*MIoU*). The values of these metrics of MAs-FC-DenseNet were significantly greater than that of other deep learning network models, including DeeplabV3+ and PSPNet, which could reach to 99.97%, 94.19%, 88.40%, 89.70%, 88.98%, and 90.14%, respectively. Moreover, the detection results of MAs-FC-DenseNet were very close to the ground truth. Thus, MAs-FC-DenseNet is a suitable model for diabetic retinopathy screening based on MA detection result.

DR is a complex, progressive, and heterogenous ocular disease associated with diabetes duration. It is generally recognized as a vascular disease like other diabetes-related diseases. Signs of DR contain the lesions such as MAs, hemorrhages, and yellowish or bright spots such as hard and soft exudates^31^. This model was designed based on the feature of MAs, but the impacts of other lesions on MA detection were not considered. In addition, DR can be classified as mild non-proliferative DR (NPDR), moderate NPDR, severe NPDR, and proliferative DR (PDR) according to disease severity^32^. Our proposed model did not consider the severity DR. The degree of DR severity should be considered as a contributing factor for MA detection in the future study.

Taken together, this study proposed a two-step model, MAs-FC-DenseNet, for the detection of MAs in FFA images, including pre-processing of FFA images by the Histogram Stretching and Gaussian Filtering and detection of MAs by the improved FC-DenseNet. This model will become a promising method for early diagnosis of diabetic retinopathy with a competitive accuracy. In the future, this model should be improved to embrace more feature-learning capacities, as well as some knowledge regarding retinal geometry and other characters.
